# Female fertility and pregnancy in autoimmune Addison’s disease – a mini review

**DOI:** 10.3389/fendo.2025.1510815

**Published:** 2025-06-18

**Authors:** Liam O’Murchadha, Agnieszka Pazderska

**Affiliations:** ^1^ Department of Endocrinology, St James’s Hospital, Dublin, Ireland; ^2^ School of Medicine, Trinity College Dublin, Dublin, Ireland

**Keywords:** Addison disease, fertility, STCA, premature ovarian insufficiency, autoimmune diseases, pregnancy

## Abstract

Autoimmune Addison’s Disease (AAD) is by far the most common cause of primary adrenal insufficiency in developed countries, occurring more commonly in women compared with men. The condition is associated with a spectrum of disorders affecting fertility and reproductive health. Premature ovarian insufficiency (POI) is a clinical condition defined by cessation of menstrual cycles and menopausal range gonadotrophins before the age of 40 years. This occurs with a prevalence of 1-2% in the general population, but has been estimated at 6-10% for women with AAD. One registry study demonstrated that one-third of those with AAD who develop POI, do so before the age of thirty. The onset of POI precedes or is contemporaneous with the diagnosis of AAD in the majority. It has also been demonstrated that women with AAD are more likely to use hormone replacement therapy. The pathophysiology of POI in this cohort is thought to be primarily through autoimmune mediated inflammation of the ovarian theca cells. In particular, cross-reacting autoantibodies to steroid-producing cells (StCA) have been identified which are present in AAD and POI. That said, when women with POI are excluded, fertility remains significantly reduced. Impaired adrenal androgenesis and resulting sex-hormone deficiency have also been implicated in subfertility in AAD. These lead to suboptimal follicular development. This, in turn, may also affect libido. Despite physiological glucocorticoid replacement therapy, patients with AAD consistently report reduced quality of life compared to matched controls. These factors may affect fecundity and likelihood of conception. Other autoimmune conditions such as hypothyroidism and type 1 diabetes occur with increased prevalence in those with AAD. These conditions have been shown to independently affect reproductive health. This review focuses on the current understanding of the factors and mechanisms impacting fertility in women with autoimmune Addison’s disease.

## 1 Introduction

Autoimmune Addison’s Disease is a life-threatening condition caused by immunological activation and attack of the adrenal cortex, resulting in deficiencies in production of adrenal steroids & their metabolites (1). It is the most common aetiology of primary adrenal insufficiency in developed countries (2) and the condition can occur in isolation or as a component of autoimmune polyendocrine syndrome types 1 (APS-1) or 2 (APS-2), the former being a monogenic disorder caused by mutations in the AIRE gene (3). In most cases, this abnormal immune response is directed at the steroidogenic enzymes of the adrenal cortex, most specifically 21-hydroxylase, antibodies to which are readily measurable in serum of affected patients (4). There is a strong genetic basis, having particular associations with HLA susceptibility loci, such as DR3-DQ2 and DR4-DQ8 (5).

The management of AAD involves lifelong replacement of glucocorticoids and mineralocorticoids, at doses attempting to approximate physiological production of these hormones. However, current steroid replacement strategies fail to adequately match physiological cortisol production and lead to under- and overexposure to cortisol at various times of the day (6). Despite advances in knowledge of the management of this condition, people with AAD have reduced quality of life (QoL) scores and increased cardiovascular risk and premature mortality (7–9).

Adrenal insufficiency of all causes may be associated with adverse effects on fertility in women of reproductive age. This may be as a result of concomitant gonadotrophin deficiency in those with pituitary disorders (10) or due to the combination of a variety of factors such as anovulation and anatomical issues for those with congenital adrenal hyperplasia (CAH) (11). However, while it has been established for more than a decade that women with autoimmune Addison’s disease are less likely to give birth (12) as indicated by lower Standardized Incidence Ratio (SIR) for birth after the diagnosis compared with before the diagnosis is made, there is a lack of robust evidence to elucidate the factors behind this. In this review, we summarize the current understanding of factors which may impact fertility & parity in women with AAD; namely premature ovarian insufficiency, the co-existence of other autoimmune disease, the potential role of diminished adrenal androgen production, psychosocial and factors related to libido and finally outcomes of pregnancy in this population group.

Below, we focus on 1) the factors pertaining to AAD which may affect female fertility and thereafter review 2) the implications of AAD for achieving a successful pregnancy.

### 1.1 Premature ovarian insufficiency

Premature ovarian insufficiency (POI) is defined as a clinical syndrome involving the cessation of menstrual cycles before the age of 40, elevation of gonadotrophins and low oestradiol (13). Most cases are idiopathic (14). POI is of clinical importance because of its effect on fertility and there is increasing evidence of its negative impact across a range of long-term health outcomes.

A recent individual patient meta-analysis of 15 observational studies (mostly prospective cohorts) reported that the risk of cardiovascular diseases was higher in women who had premature menopause compared with those whose menopause was at the age of 50-51 years (15). Women with POI are more likely than the general population to experience sexual dysfunction and depression (16). POI has been shown to be associated with lower bone mineral density scores compared with women who have menopause >40 years (17) and there is some evidence of increased rates of cognitive decline in women with POI (18).

The pooled prevalence of POI in the general population has been estimated in a recent meta-analysis of 31 studies (19) at as 3.7% (95% confidence interval: 3.1-4.3), with higher prevalence in low to medium Human Development Index countries. It has been reported that autoimmunity is implicated in 5 to 30% of cases of POI.

Premature ovarian insufficiency and early menopause occur more frequently in patients with primary adrenal insufficiency with estimates of 6-20% in this population (20–22). An observational population-based cohort study of the Norwegian National Addison Registry examined the records of 461 women with autoimmune Addison disease (23) and reported a prevalence of POI of 10.2%. One-third of these women developed POI before 30 years of age. Interestingly, in this cohort women with POI were more likely to have a longer duration of AAD than those without POI (26.8 vs 20.1 years, *P* = .003). There was a strong positive correlation between age at diagnosis of AAD and age at menopause (*P* <.001). It had previously been reported that even for AAD patients without POI, the median age of menopause was almost four years younger than for the general population (24).

Women with AAD because of autoimmune polyendocrine syndrome type 1 (APS-1) are reported to be at highest risk of developing POI, in excess of 40% (20).

The underlying mechanism to explain the high prevalence of POI in women with AAD is due to autoimmune oophoritis, itself due to the presence of autoantibodies against steroid producing cells (StCA) (25, 26). Steroidogenic enzymes are expressed in both the ovary and adrenal cortex. StCA antibodies are found in the majority of women with both conditions and the most important include steroid 17α-hydroxylase (17α-OH) and cytochrome P450 side-chain cleavage enzyme (P450scc) (27). Falorni (25) studied 57 patients with POI without adrenal insufficiency and 24 women with POI in association with AAD. In that study, 87% of women with POI and AAD had measurable steroid-cells antibodies, compared to none in the POI without associated adrenal insufficiency cohort.

The potential for using measurements of StCA to predict later developed of POI could be of clinical utility. De Ballis (28) followed 33 women under the age of 40 years with AAD, but without clinically overt POI, annually for a ten-year period to investigate the predictive capacity of StCA for the development of overt ovarian dysfunction. All women in the group with StCA at high titer at the start of the study (>1:32) developed ovarian failure in the follow up period, whereas none of the women without StCA at baseline either developed these antibodies or developed menstrual dysfunction over the duration of the study period. Such evidence raises the promise of measuring StCA in women with AAD to help clinically guide those patients who might most benefit from fertility preservation treatment.

Erichsen et al. (22) used Standardized Incidence Ratio (SIR) for birth to assess changes in fertility & parity before and after a diagnosis of AAD. The SIR for birth before the women were diagnosed was 0.97 (CI, 0.845–1.095). After AAD diagnosis, this SIR fell to 0.69 and even when the subgroup with POI was excluded the number of observed births remained well below the expected rate, with an SIR of 0.72. Clearly, such results would point to other factors in play in reduced fertility in women with AAD, beyond POI.

It must be pointed out that, to date, there is no reliably effective way of restoring ovarian function for these women. POI likely presents as a spectrum of disease, with some women achieving a successful pregnancy without any specific treatment. Van Kasteren (29) reported in 1999, that women with POI still have a 5-10% chance of conceiving after the diagnosis is established.

There has, though, been increasing interest in evaluating interventions which might preserve ovarian function for longer. One randomized controlled trial (30) assessed DHEA supplementation versus placebo across several parameters. At the end of the treatment period, there was no significant difference in AMH concentrations, FSH levels or return of menses between the two groups. There was a higher antral follicle count (AFC) observed in the DHEA group, but the clinical significance of this in such a small study group (n=22) is unclear. Intraovarian injection of autologous platelet rich plasma (PRP) has been investigated in two observational studies (31, 32). In both studies, transvaginal PRP lead to a statistically significant increase in AMH and antral follicle count. In the former, 7% of patients achieved spontaneous pregnancy after PRP treatment. Randomized controlled trials to evaluate this intervention are clearly required, before one can comment on its effectiveness.

Finally, there are isolated reports of restoration of ovarian function in those being treated with immunomodulatory interventions for other indications. One such notable example (33) concerns a woman with AAD who had a 13-year history of POI, demonstrated by amenorrhea, hypergonadotrophic hypogonadism and evidence of StCA in the serum. Ovarian biopsy confirmed autoimmune oophoritis. Following eight months of treatment with azathioprine for ulcerative colitis, the woman had spontaneous return of menses and became pregnant shortly thereafter. Such cases, though rare, demonstrate the potential of immunomodulatory medications to alter the course of POI, even for women with longstanding amenorrhea.

### 1.2 Adrenal androgen production

Autoimmune Addison’s Disease also results in deficiencies of adrenal androgens and their precursors, particularly DHEAS from the zona reticularis (34). However, unlike glucocorticoids and mineralocorticoids, androgens are not routinely replaced in clinical practice. There is a progressive age-related decline in DHEA concentrations in the healthy female population (35). In both primary and secondary adrenal insufficiency, it has been well established that (36–38) serum testosterone and related precursors are significantly reduced compared to those without the condition. Study of this topic in women is complicated by the fact that commercially available testosterone assays show divergent results when analyzing the low androgen levels typically seen in women compared with men (39).

Androgens are thought to play key roles in the regulation of ovarian follicular development. Follicles are the basic physiological unit, enabling reproduction. This has been revealed in animal models (40, 41) where knockout mice lacking the androgen receptor had reduced fertility, impaired folliculogenesis, reduced ovulation and increased rates of POI. Sen et al. (42) have demonstrated, again in mice, that androgens are promotors of follicular development by regulating the balance between follicular growth and apoptosis. This is achieved by suppressing follicular atresia by inducing the expression of miR-125b, a microRNA which suppresses proapoptotic protein synthesis and by enhancing follicular growth by upregulating FSH expression.

Lower than normal serum androgen concentrations observed in adrenal insufficiency might well suggest hypoandrogenism as a significant factor in the observed subfertility after the diagnosis of AAD, however no studies have examined this to date.

### 1.3 Psychosocial factors & libido

A number of studies have established the effects of circulating androgens on sexual motivation and responsiveness in women (43–45). Jacobsen et al. demonstrated, in a cohort of 560 healthy women aged 19-65 years, both free testosterone and androstenedione correlated well with measures of sexual desire. In a subgroup of women aged 25-44, total testosterone and DHEAS also correlated with the measures of sexual desire.

Knowing that women with AAD have lower androgen concentrations, it is plausible that sexual motivation and function would be adversely impacted in AAD, but clearly libido is a complex and subjective concept. It may be thought of as the culmination of a multiple hormonal, non-hormonal and environmental inputs. The degree to which AAD effects sexual function in women is not well established, with conflicting results reported by studies.

The most complete picture, to date, is provided by Erichsen et al. (22), who specifically examined sexual function in women with autoimmune adrenal insufficiency using the Sexual Activity Questionnaire (SAQ) (46), which looks at three areas; relationship status, reasons for sexual inactivity, and sexual functioning. In the study, 148 women with AAD did not report a reduction in sexual activity compared with 740 age-matched controls. In fact, women with AAD reported that they were comparatively more sexually active, had higher SAQ scores for sexual pleasure and reduced sexual discomfort; the precise reasons for these findings are not clear.

A smaller study (47) aimed to examine the prevalence of female sexual dysfunction in primary adrenal insufficiency using the Female Sexual Function Index-6 (FSFI-6), which unlike SAQ includes female sexual desire among other parameters. The other parameters are arousal, lubrication, orgasm, satisfaction and dyspareunia. They identified that women with AAD report significantly increased sexual issues compared with the control group (68.2% versus 8.7%). Specifically, adverse differences in AAD for arousal, desire and sexual satisfaction were reported.

Most recently, the DREAM (48) group investigating dual release versus conventional hydrocortisone treatment reported data on sexual function in adrenal insufficiency (primary and secondary aetiologies), showing that 94% of the patients exhibited diminished sexual desire, however no associations were observed between sexual function and sex steroid levels. At the end of the intervention period, there was no significant difference in Female Sexual Function Inventory (FSFI) scores in either treatment group. For pre-menopausal women, rates of sexual activity seen were similar to that previously reported for healthy populations, but rates were significantly reduced in the postmenopausal group; the authors postulate their findings imply that for postmenopausal women specifically, there is a comparatively greater physiological role for adrenal androgens in sexual motivation relative to ovarian androgens.

Reduced quality of life (QoL) scores are consistently reported by women with AAD, despite adequate glucocorticoid and mineralocorticoid replacement (7). The effect of reduced QoL on libido and sexual function in this specific population has not been studied.

In summary, questions relating to sexual function in AAD have not been satisfactorily answered to date. Despite low androgens being associated with reduced sexual desire in the general population, such correlations in adrenal insufficiency have not been consistently demonstrated.

### 1.4 Co-existence of other conditions

Women with AAD are at much higher risk of developing other autoimmune endocrinopathies (3, 49). Addison’s disease is a major component of the autoimmune polyendocrine syndromes APS-1 and APS-2. While AAD can occur in isolation, it is associated with the presence of other autoimmune conditions in 50–80% of cases. Current estimates are that of all people with autoimmune Addison’s disease, about two-thirds have an autoimmune polyendocrine syndrome with APS Type 1 accounting for 15% of people with AAD (50). These co-existing autoimmune diseases include autoimmune thyroid disease, type 1 diabetes, gastritis, POI, vitiligo and hepatitis. Thyroid disease (51) and type 1 diabetes (52) are both independently associated with reduced fertility. Thyroid autoimmunity appears to confer an increased risk of miscarriage even with biochemical euthyroidism. One study has identified adverse effects of type 1 diabetes on fertility even before the woman is diagnosed with diabetes (53). Lin et al. have additionally reported in their study of Taiwanese women that type 1 diabetes in association with thyroid disease had a further negative impact on live birth rate.

It seems plausible therefore that the high prevalence of additional diseases seen concurrently with AAD has a contribution to the reduction seen in some aspects of fertility. That said, Bjornsdottir (12) showed in a subgroup analysis that women with isolated AAD also did have a lower parity.

## 2 Pregnancy

**Figure 1:**
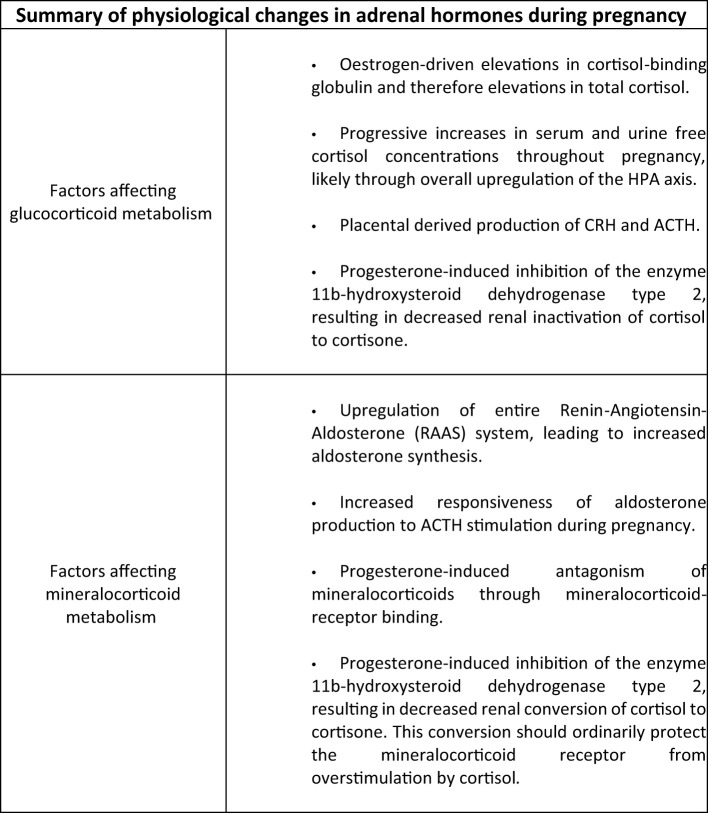
Summary of physiological changes in pregnancy relevant to Addison’s Disease.

During pregnancy a number of physiological changes in the HPA axis and mineralocorticoid status occur in women without adrenal insufficiency (**Figure 1**). Firstly, alterations in glucocorticoid metabolism have been demonstrated in a study of plasma and urinary cortisol in pregnancy and the peripartum period (54). It has previously been reported (55) that increases in oestrogen during pregnancy drive elevations in cortisol-binding globulin and therefore elevations in total cortisol. However, Jung et al, in this longitudinal study (54), also demonstrated progressive elevations in plasma free cortisol concentrations and urinary free cortisol measurements through the course of the pregnancy, compared with non-pregnant controls. They found that mean serum free cortisol concentrations were 1.2-, 1.4-, and 1.6-fold elevated during the first, second, and third trimesters, respectively, compared with the control group and that urinary free cortisol concentrations also became progressively elevated, peaking in the third trimester with levels 3.1 times that seen in the non-pregnant group. These results suggest upregulation of the hypothalamic-pituitary-adrenal axis during pregnancy. The placenta was identified as a source of ACTH and CRH in the 1980s (56) and it has recently been discovered (57) that the human placenta releases CRH mRNA packaged within extracellular vesicles into the maternal blood. During pregnancy, CRH appears to play a role in regulation of pregnancy duration with elevated levels occurring in women who deliver preterm, while a lower concentration is associated with an extended length of pregnancy (58).

Mineralocorticoid metabolism during pregnancy is also altered in those with normal HPA axis activity. The entire renin-angiotension-aldosterone pathway is upregulated leading to elevations in aldosterone concentrations (59, 60). One study reported serum aldosterone rise from 8.0 ng/dl in the first trimester to 25.3 ng/dl during the third trimester of pregnancy (*P* = 0.001) and also demonstrated increased responsiveness of aldosterone production to ACTH (61). In addition, progesterone-induced inhibition of the enzyme 11β-hydroxysteroid dehydrogenase type 2 in the kidney results in decreased renal inactivation of cortisol to cortisone, and subsequently an increased activation of the mineralocorticoid receptor by excess cortisol (62).

The changes in gluco- and mineralocorticoid metabolism have important clinical consequences for women with AAD during pregnancy. Prior to the availability of reliable steroid replacement preparations, maternal mortality was reported to be as high as 35% with 1/3 of infants dying at time of delivery (63). Pregnant women with AAD may be at higher risk of developing adrenal crisis due to for example; hyperemesis during the first trimester or insufficient parenteral glucocorticoid in the puerperium (64). Current guidance emphasizes the need to take account of the increased physiological cortisol production during pregnancy described above, and that an increase in hydrocortisone dose of 20-40% should be implemented, especially in the final trimester (65). An increased dose of fludrocortisone during the latter stages of pregnancy is also frequently recommended, though robust evidence is lacking.

The outcome of pregnancies in women diagnosed and appropriately treated for AAD has typically been considered as favorable (66). However, a Swedish Registry Study from 2010 (12), found that the diagnosis of AAD was still a risk factor for adverse pregnancy events. This study compared 1118 women aged 15–47 during the period 1973 to 2006, who had a diagnosis exclusively of AAD with age matched women without AAD. Compared with controls, the odds of having childbirth were not significantly reduced in women before the AAD diagnosis but were significantly reduced after the diagnosis was made. The odds of having three or more infants were three times lower among women after their AAD diagnosis compared with controls. No women were diagnosed with AAD while pregnant. Infants delivered 3 years or less before the date of the mother’s diagnosis of AAD were more often born preterm (adjusted OR, 2.40; 95% CI, 1.27–4.53). The closer to the AAD diagnosis that the mother delivered, the higher was the risk of preterm delivery, with a five times increased risk 1 yr or less before the mother’s diagnosis of AAD. Risk of low birth weight was more than three times higher among infants delivered 3 yr or less before the mother’s diagnosis of AAD (adjusted OR, 3.50; 95% CI, 1.83–6.67). Compared with the control group, the risks of caesarean (adjusted OR, 2.35; 95% CI, 1.68–3.27) and preterm delivery (adjusted OR, 2.61; 95% CI, 1.69–4.05) were more than doubled among mothers diagnosed with AAD. Birth weight did not differ between the two groups. There was no statistically significant increased risk of maternal complications observed and the authors stressed that the majority of both diagnosed and undiagnosed AAD ultimately had “normal” pregnancies with infants delivered vaginally, at full term without complications. They also highlighted that physician preference may have been a potential explanation for the increased caesarean section rate.

More recently, Bothou et al. (67), retrospectively analyzed pregnancy outcomes in 113 women with adrenal insufficiency, 44% with AAD. 15 of 55 women with AAD had at least one miscarriage previously, which was significantly lower than for women with CAH. 8.9% of women had an adrenal crisis during the course of their pregnancy. 16% had a pre-term delivery, which was lower than for both CAH and secondary adrenal insufficiency (32% and 21% respectively).

Schneiderman et al. (68) performed a retrospective cohort study of data pertaining to the pregnancies of almost 8 million women in the USA between 2003 and 2011, 552 of which had adrenal insufficiency. It is important to point out that as this study utilized ICD codes to capture relevant patient data, it has included women with other forms of primary adrenal insufficiency (and potentially secondary AI) besides AAD. There was an increased rate of caesarean section and preterm delivery in the adrenal insufficiency group, though these women were also significantly older that the control population. Contrary to the data from earlier studies above, women with adrenal insufficiency were found to be at higher risk of a variety of adverse maternal outcomes, including premature rupture of the membranes and post-partum infections. Adrenal insufficiency was associated with a statistically significant increase in overall mortality risk with three deaths among 552 patients (OR 22.30, 95% CI 6.82–72.96). This represents a mortality rate of 0.5% for women with AAD in this study. Impaired fetal growth and increase congenital abnormalities were also identified.

These few studies offer some conflicting information on pregnancy and fetal outcomes for women with AAD. The increased rate of caesarean section does seem to be a somewhat consistent finding, though the reasons for this are likely to be complex and multifactorial. There is also evidence of increased risk of premature birth, which would fit well with the postulated role of CRH as one regulator of pregnancy duration. However, in another smaller retrospective questionnaire study of 54 women (69), the investigators did not find any increase in preterm delivery for women with AAD. These conflicting findings reinforce the need for further large, well designed prospective studies of pregnancy outcomes with AAD.

## 3 Conclusions

In summary, there are many knowledge gaps in our understanding of the extent to which AAD affects female sexual function and reproduction. It is well established that women with AAD are at higher risk of POI, but the literature would suggest that fertility remains significantly reduced, beyond the obvious effects of POI.

While observational studies have reported increased risk of preterm birth and caesarean section, it is still the case one can reassure pregnant women with AAD that the vast majority with well controlled disease will have uneventful pregnancies with no major maternal complications.

There are many potential factors that contribute to the observed reductions in parity after women are diagnosed with AAD. These include the role of androgen deficiency, the increased prevalence of other conditions which may independently have an effect and the impact observed on overall quality of life. However, these theories have not been consistently borne out by published literature to date and there remains many conflicts and contradictions to resolve so that we can provide comprehensive reproductive healthcare to our patients with autoimmune Addison’s disease.
